# Peroxiredoxin 5 deficiency exacerbates iron overload-induced neuronal death via ER-mediated mitochondrial fission in mouse hippocampus

**DOI:** 10.1038/s41419-020-2402-7

**Published:** 2020-03-23

**Authors:** Dong Gil Lee, Min Kyoung Kam, Sang-Rae Lee, Hong Jun Lee, Dong-Seok Lee

**Affiliations:** 1 School of Life Sciences, BK21 Plus KNU Creative BioResearch Group, Kyungpook National University, Daegu, 41566 Republic of Korea; 2 National Primate Research Center, Korea Research Institute of Bioscience and Biotechnology (KRIBB), Cheongju, 28116 Republic of Korea; 3 College of Medicine, Chungbuk National University, Chungbuk, Republic of Korea; 4 Department of Radiology, Chungbuk National University Hospital, Chungbuk, Republic of Korea; 5 Research Institute, e-biogen Inc., Seoul, 07282 Korea

**Keywords:** Iron, Cell death in the nervous system, Molecular neuroscience

## Abstract

Iron is an essential element for cellular functions, including those of neuronal cells. However, an imbalance of iron homeostasis, such as iron overload, has been observed in several neurodegenerative diseases, including Alzheimer’s disease and Parkinson’s disease. Iron overload causes neuronal toxicity through mitochondrial fission, dysregulation of Ca^2+^, ER-stress, and ROS production. Nevertheless, the precise mechanisms between iron-induced oxidative stress and iron toxicity related to mitochondria and endoplasmic reticulum (ER) in vivo are not fully understood. Here, we demonstrate the role of peroxiredoxin 5 (Prx5) in iron overload-induced neurotoxicity using Prx5-deficient mice. Iron concentrations and ROS levels in mice fed a high iron diet were significantly higher in *Prx5*^*−/−*^ mice than wildtype (WT) mice. Prx5 deficiency also exacerbated ER-stress and ER-mediated mitochondrial fission via Ca^2+^/calcineurin-mediated dephosphorylation of Drp1 at Serine 637. Moreover, immunoreactive levels of cleaved caspase3 in the CA3 region of the hippocampus were higher in iron-loaded *Prx5*^*−/−*^ mice than WT mice. Furthermore, treatment with N-acetyl-cysteine, a reactive oxygen species (ROS) scavenger, attenuated iron overload-induced hippocampal damage by inhibiting ROS production, ER-stress, and mitochondrial fission in iron-loaded *Prx5*^*−/−*^ mice. Therefore, we suggest that iron overload-induced oxidative stress and ER-mediated mitochondrial fission may be essential for understanding iron-mediated neuronal cell death in the hippocampus and that Prx5 may be useful as a novel therapeutic target in the treatment of iron overload-mediated diseases and neurodegenerative diseases.

## Introduction

Iron is an essential element for cellular functions, including those of neuronal cells. Some of these vital functions are DNA synthesis, mitochondrial respiration, production of neurotransmitters, and myelin formation^1–3^. However, an imbalance of iron homeostasis can produce reactive oxygen species (ROS), which can damage lipids, proteins, and DNA^4,5^. Moreover, several studies have reported that iron accumulation in the brain plays a role in various neurodegenerative disorders, such as Alzheimer’s disease, Parkinson’s disease, and Huntington’s disease, and that iron accumulation causes mitochondrial dysfunction, oxidative damage, and inflammation^6,7^. But, the precise mechanisms underlying iron overload-induced neurotoxicity are not fully understood.

In biological systems, iron participates in the production and metabolism of ROS. If free iron is increased by iron accumulation, then it can catalyze the decomposition of hydrogen peroxide (H_2_O_2_) to form hydroxyl radicals (HO˙) via Fenton’s reaction^8,9^. Iron-induced ROS can cause ryanodine receptor-mediated calcium release and damage to biomolecules such as lipids, DNA, and proteins, thereby leading to neurodegeneration^10,11^. Peroxiredoxins (Prxs), which are a family of antioxidant enzymes, can decrease oxidative stress by eliminating H_2_O_2_ and by participating in various intracellular oxidative signaling pathways^12–14^. Moreover, overexpression of Prxs provides a protective effect in various cells, including neuronal cells, whereas knockdown of Prxs makes these cells more sensitive to oxidative stress-induced cell death^15–17^. Prxs have been reported to play an important role in redox-sensitive signaling. In previous studies, we have shown that Prx5 exerts a protective effect against iron overload-induced neuronal cell death by inhibiting mitochondrial fission and endoplasmic reticulum (ER)-stress in hippocampal HT-22 cells^18^. However, the relationship between Prx5 and iron neurotoxicity in vivo has not been elucidated to date.

Mitochondria are essential organelles and are highly dynamic. Mitochondria consistently fuse and divide through the processes of fusion and fission, respectively. An imbalance in mitochondrial dynamics is associated with neuronal loss in neurodegenerative disorders^19–21^. Dysregulated iron homeostasis is known to cause mitochondrial DNA damage and loss of respiratory capacity^22^. In addition, various neurodegenerative diseases are highly related to iron dysregulation and mitochondrial dysfunction^23^. Recent studies report that the ER mediates mitochondrial fission^24,25^. Because iron overload is known to induce ER-stress in rat and mouse models and ER-stress has been associated with morphological changes in mitochondria^26–28^, we investigated how iron overload induces ER-stress and ER/Drp1-mediated mitochondrial fission in the murine hippocampal cell line HT-22^18^. However, it is unknown whether these events occur in iron-loaded mouse brain in vivo.

In this study, we investigated the mechanism of iron overload-induced toxicity on the brain in iron-loaded mice. We further evaluated the effect of Prx5 against iron overload-induced neurotoxicity related to ER-mediated mitochondrial fission in iron-loaded Prx5-deficient mice.

We concentrated on understanding the mechanism of iron neurotoxicity to contribute to the development of therapies for iron overload-related disorders.

## Materials and methods

### Materials

Ferric ammonium citrate (FAC) and N-acetyl-cysteine (NAC) were obtained from Sigma (St. Louis, MO, USA). BAPTA-AM were obtained from Invitrogen (Carlsbad, CA, USA).

### Animal preparation

Wildtype (WT) and Prx5-deficient (*Prx5*^*−/−*^) mice with a C57BL/6 background were maintained under the guidelines of the Institutional Animal Care and Use Committee of the Kyungpook National University (Daegu, Korea). Animals were kept under standard environmental conditions; a temperature of 20–22 °C, humidity of 50–60%, light/dark cycles of 12 h each condition, and free access to food and water. Males (4-week old; *n* = 3–4 per group) were used in these studies.

WT and *Prx5*^*−/−*^ mice were segregated randomly into two groups that were fed either a 25 g iron/kg high iron diet (HFe; Central Lab. Animal Inc., Seoul, Korea) or a standard diet (ND; AIN-76A, Central Lab) for 8 weeks. Iron-loaded mice were further divided into two subgroups that were given ad libitum access to water alone or water with NAC. Water was switched to 2 mM/L of NAC at 3 weeks before the terminal experiment for the NAC treatment group. The NAC dose given considered differences in rodent and human metabolism and was chosen to deliver a dose range (326–570 mg/kg/day) to the mice that is equivalent to the human dose of 20–30 mg/kg/day. The body weights of mice in each experimental group were measured once a week.

### Measurement of iron concentration

Total iron content was measured using an Iron Assay Kit (#MAK024, Sigma). Briefly, 10 mg Hippocampal tissues were homogenized in Iron Assay buffer. The homogenized tissues were centrifuged at 13,000 × *g* for 10 min at 4 °C, and supernatant was used in assay. Then, iron assay was performed according to the manufacturer’s instructions.

### Genotyping

Mouse DNA was extracted from the tails of WT and *Prx5*^*−/−*^ mice. The terminal 2–5 mm of each tail was collected and placed directly into an Eppendorf tube. Lysis reagent (1 ml; Viagen, LA, USA) containing 0.5 mg/ml proteinase K (Sigma) was added to the tube and incubated at 55 °C for 5–6 h or until no tissue clumps were observed. Hairs were removed by centrifuging for 1 min and transferring the supernatant to another tube. To inactivate proteinase K, the supernatant was incubated at 85 °C for 45 min in a water bath. To obtain pure DNA, 250 mM NaCl and isopropanol (70% of the total volume) were added to each sample and incubated for 1 min at room temperature. Samples were centrifuged at 13,000 rpm for 10 min, and the supernatants were removed. EtOH (1 ml, 80% v/v) was added to wash the pellet, and the sample was then centrifuged at 13,000 rpm for 10 min. Supernatants were removed, and pellets were resuspended in distilled H_2_O.

The primer sequences used for PCR are as follows: endogenous Prx5 forward (5′-ATTCTTTGGTGTCTCTCTTTGGG-3′), neomycin forward (5′-CCCGTCATATTGCTGAAGAGC-3′), and Prx5 reverse (5′-CTTCACTTTCTCCTCCAAATCCC-3′). PCR was performed with an initial denaturation at 94 °C for 3 min, followed by 30 cycles of amplification (94 °C for 40 s, 58 °C for 30 s, 72 °C for 1 min) and a final extension at 72 °C for 3 min.

### Primary hippocampal neuron-enriched culture

Hippocampi were dissected from fetal C57BL/6 (WT and *Prx5*^*−/−*^) mice at 18–19 days gestation and placed in Hank’s balanced salt solution (HBSS, Welgene). The hippocampi were gently minced with a sterile scissor in sterile HBSS containing 15 mM HEPES, 10 mM sodium bicarbonate, and 1% penicillin/streptomycin. The minced hippocampal tissues were transferred to 15 ml conical tubes and centrifuged at 600 rpm for 5 min. The supernatant was removed, and 1 ml of 0.25% Trypsin/EDTA solution (Welgene) was added to the hippocampal tissues. Tissues were gently dissociated by pipetting using a narrow tip and incubated at 37 °C for 5 min. DNase I solution (100 units/ml) was then added to each conical tube and incubated at 37 °C for 1 min. Samples were centrifuged at 600 rpm for 5 min, and supernatants were carefully removed. The hippocampal neurons were resuspended in 5 ml of plating medium (DMEM/F12 containing 15 mM HEPES, 45 mM NaHCO_3_, 0.5 mM sodium pyruvate, 1X nonessential amino acid, and N-2 supplement). The suspended hippocampal neurons were plated on poly-L-lysine (Sigma)-coated 6-well plates (SPL, Pocheon, Korea) at a density of 1.0 × 10^6^ cells/well in 2 mL of plating medium and grown in a humidified 5% CO_2_ incubator at 37 °C. Less than 12 h after plating, the medium in each well was replaced with 2 mL of neurobasal medium (Invitrogen) containing 1X glutamax (Welgene) and B-27 supplements. Hippocampal neurons were used for experiments after 7 days in culture.

### Measurement of tissue ROS levels

We collected the hippocampus from each WT and *Prx5*^*−/−*^ mouse. Hippocampal tissue lysates were prepared using ice-cold PRO-PREP protein extraction solution (iNtRON Biotechnology, Seongnam, Korea). All lysates were centrifuged for 10 min at 13,000 rpm, and then supernatants were kept frozen (−80 °C) until analysis. Tissue ROS analysis was performed using an OxiSelect In Vitro ROS/RNS Assay Kit (Cell Biolabs, USA), according to the manufacturer’s instructions.

### Measurement of intracellular ROS and Ca^2+^ levels

After hippocampal neuron cells (1 × 10^6^) were cultured for 7 days in 6-well plates, cells were treated with FAC for 48 h. The cells were then harvested by trypsinization. For measuring intracellular ROS and calcium levels, the harvested cells were washed with PBS and then incubated with 2.5 μM of CM-H2DCFDA (Thermo Fisher Scientific) and Fluo-4 AM (Thermo Fisher Scientific) for 15 min at 37 °C. The cells were then washed twice with PBS and analyzed by flow cytometry (FACSverse; BD Biosciences).

### Iron and H&E staining

Following HFe feeding for 8 weeks, the hippocampi of mice were isolated from WT and *Prx5*^*−/−*^ mice. Hippocampal tissues were fixed in 4% paraformaldehyde (Merck, Darmstadt, Germany) overnight, embedded in paraffin, and cut into 5 μm sections, which were then stained with a hematoxylin and eosin (H&E) kit and an iron stain kit (Abcam) using standard protocols. The iron stain kit is based on the Prussian blue reaction in which ionic iron reacts with acid solutions of ferrocyanides to produce a blue color.

### RNA extraction and real-time PCR (RT-qPCR)

Total RNA was isolated from hippocampal tissues of WT and *Prx5*^*−/−*^ mice using TRI solution (Bio Science Technology, Gyeongsan, Korea) according to the manufacturer’s instructions. The cDNA was synthesized using 1 μg of each total RNA and AccuPower RT-PCR Premix (Bioneer).

The primer sequences used for PCR are as follows: mouse H-ferritin (Forward: 5′- GACCGTGATGACTGGGAGAG-3′; Reverse: 5′-TAGCCAGTTTGTGCAGTTCCA-3′), mouse L-ferritin (Forward: 5′-ATGGGCAACCATCTGACCAA-3′; Reverse: 5′-TTGAGAGTGAGGCGCTCAAA-3′), and mouse transferrin receptor (Forward: 5′-TCCGCTCGTGGAGACTACTT-3′; Reverse: 5′-ACATAGGGCGACAGGAAGTG-3′).

Comparative real-time PCR using the ΔΔCT method was performed with a StepOnePlus real-time PCR system and Power SYBR green PCR master mix (Thermo Fisher Scientific). PCR conditions were: initial denaturation at 95 °C for 30 s, followed by 40 cycles of amplification (95 °C for 5 s, 60 °C for 30 s).

### Western blot analysis

Whole protein lysates were prepared using ice-cold PRO-PREP protein extraction solution (iNtRON Biotechnology, Seongnam, Korea), and protein concentrations were measured using the Bradford assay (Bio-Rad, Hercules, CA, USA). Lysates (20–30 μg) were separated on 10–15% sodium dodecyl sulfate-polyacrylamide gradient gels. The proteins were then transferred onto nitrocellulose membranes (BD Biosciences, Franklin Lakes, CA, USA). The membranes were blocked with 5% skim milk (BD Biosciences) and incubated overnight at 4 °C with the following primary antibodies: anti-Ferritin heavy chain (#sc-376594, Santa Cruz,CA, USA), anti-Ferritin light chain (#sc-390558, Santa Cruz), anti-Transferrin receptor (#AB84036, Abcam, Cambridge, MA, USA), anti-ATF6 (#sc-166659, Santa Cruz), anti-p-IRE1α (#AB48187, Abcam), anti-Prx5 (#LF-PA0210, AbFrontier, Seoul, Korea), anti-Drp1 (#sc-32898, Santa Cruz), anti-GADD34 (#sc-8327, Santa Cruz), anti-β-actin (#4970, Cell Signaling, Danvers, MA, USA), anti-CHOP (#2895, Cell Signaling) anti-IRE1α (#3294, Cell Signaling), anti-Bip (#3177, Cell Signaling), anti-eIF2α (#9722, Cell Signaling), anti-p-eIF2α (#3597, Cell Signaling), anti-p-Drp1(Ser616) (#3455, Cell Signaling), anti-p-Drp1(Ser637) (#4867, Cell Signaling), anti-cleaved caspase3 (#9661, Cell Signaling), and anti-pan-calcineurin A (#2614, Cell Signaling). The membranes were washed five times with 10 mM Tris-HCl (pH 7.5) containing 150 mM NaCl and 0.1% Tween-20 (TBST) and incubated with horseradish peroxidase-conjugated goat anti-rabbit and anti-mouse IgGs (Thermo Fisher Scientific) for 1 h at room temperature. After removing excess secondary antibodies, the membranes were washed six times with TBST, and specific binding was detected using Clarity Western ECL substrate (Bio-Rad) according to the manufacturer’s instructions.

### Cell viability assay

Hippocampal neuron cells (1 × 10^4^) were cultured in 12-well plates for 24 h, and then FAC was added. Cell viability was assessed using 3-(4,5-Dimethyl-2-thiazolyl)-2,5-diphenyl-2H-tetrazolium bromide (MTT; Sigma). The culture medium in each well was removed carefully and then replaced with 0.5 mg/mL MTT solution dissolved in phenol red-free DMEM for 30 min at 37 °C. The medium was removed, and 100 μL of DMSO was added to each well to dissolve the formazan crystals. Absorbance was measured at 550 nm using an infinite-F50 microplate reader (TECAN, Switzerland).

### Immunocytochemistry

For imaging Drp1, mitochondria, and ER, hippocampal neuron cells were seeded onto 0.1% poly-D-lysine-coated 24-mm round coverslips (Marienfeld, Germany) and incubated for 7 days. After treatment with FAC, cells were fixed with 4% paraformaldehyde (Sigma) in PBS, permeabilized with 0.25% Triton X-100 in PBS (PBST) for 10 min, and treated with 1% bovine serum albumin in PBST. Cells were incubated overnight at 4 °C with anti-Drp1 antibody diluted in blocking solution, followed by incubation with Alexa Fluor 405 goat anti-rabbit secondary antibody (Thermo Fisher Scientific) for 2 h at room temperature. Cells were washed twice with PBS and then stained using MitoTracker Red and ER-Tracker Green (Thermo Fisher Scientific). Cells were washed twice with PBS, and coverslips were mounted on the slides using VECTASHIELD mounting medium (Vector Laboratories). Images were obtained using Carl Zeiss LSM-710 confocal microscope (Carl Zeiss). The quantitation of co-localization was measured by ImageJ software^29^.

### Immunohistochemistry

Before separating tissues from each mouse, perfusion was carried out. After separating tissues from mice, all tissues were fixed at 10% formalin in 4 °C overnight. Tissues were cryosectioned with an HM525 NX Cyrostat (Thermo Scientific). Sectioned tissues were incubated with an anti-NeuN (#94403, Cell Signaling), anti-GFAP (#sc-166458, Santa Cruze) and anti-cleaved caspase3 antibodies (#9661, Cell Signaling) at 4 °C overnight. Tissues were then incubated with Alexa 488 goat anti-mouse and Alexa 555 goat anti-rabbit secondary antibody (Thermo Scientific) at 4 °C overnight. Images were obtained using an LSM-710 confocal microscope (Carl Zeiss).

### Statistical analysis

The data represent as the mean ± SEM of individual samples. Experimental differences were tested for statistical significance using one-way or two-way ANOVA with GraphPad Prism 5 software (San Diego, CA, USA). A *p*-value of < 0.05 was deemed to be statistically significant and is indicated on graphs by an asterisk. *p*-values of < 0.01 and < 0.001 are indicated by two and three asterisks, respectively.

## Results

### Prx5 deficiency exacerbates iron overload in the hippocampus

**Fig. 1:**
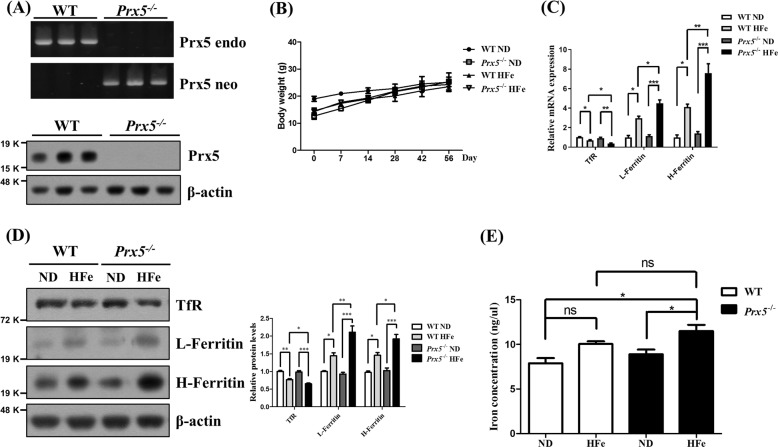
Prx5 deficiency exacerbated iron overload in the hippocampus. **a** Genotyping of Prx5 genes (upper panel) and the protein levels of Prx5 (bottom panel) in WT and *Prx5*^*−/−*^ mice (*n* = 3). **b** The graph shows the body weight of WT and *Prx5*^*−/−*^ mice (*n* = 6) with or without a high iron diet (HFe). **c** The mRNA levels of H-ferritin, L-ferritin, and transferrin receptor were evaluated by RT-qPCR in hippocampal tissues of WT and *Prx5*^*−/−*^ mice (*n* = 4) with or without HFe. **d** The expression levels of H-ferritin, L-ferritin, and transferrin receptor were evaluated by western blot analysis in hippocampal tissues of WT and *Prx5*^*−/−*^ mice (*n* = 3) with or without HFe. The graph shows the quantification of protein/β-actin. **e** The levels of iron concentration were measured by an iron assay kit in hippocampal tissues of WT and *Prx5*^*−/−*^ mice (*n* = 4) with or without HFe. Data are expressed as mean ± SEM of three independent experiments. ns *p* > 0.05, *p < 0.05, **p < 0.01, and ***p < 0.001.

**Fig. 2:**
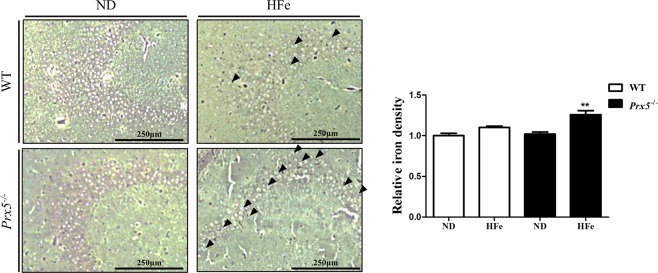
Iron staining in hippocampi of WT and Prx5^−/−^ mice with or without HFe. Hippocampi of WT and *Prx5*^*−/−*^ mice (*n* = 3) with or without HFe were stained by an iron stain kit and observed by optical microscope. Stained iron deposits (blue) and counterstained nuclei (red). The graph shows the relative density of iron (blue); scale bar = 250 μm. Data are expressed as mean ± SEM of three independent experiments. **p < 0.01.

To investigate iron overload-induced neurotoxicity in the hippocampus, we used a high iron diet (HFe) in WT and *Prx5*^*−/−*^ mice. Before feeding HFe, we confirmed genotype and Prx5 protein expression of WT and *Prx5*^*−/−*^ mice using genotyping and western blot analysis. The results showed that Prx5 genes and protein levels are successfully deleted in *Prx5*^*−/−*^ mice (Fig. 1a). Next, we investigated the change in body weight after feeding ND and HFe in WT and *Prx5*^*−/−*^ mice. Changes in body weights were negligible in WT and *Prx5*^*−/−*^ mice with or without HFe (Fig. 1b). Subsequently, we investigated iron overload in hippocampi of WT and *Prx5*^*−/−*^ mice with or without HFe. Several studies reported that excess iron increases iron storage proteins, such as ferritin, and decreases transferrin receptor (TfR) by mRNA degradation via the inactivation of iron-responsive elements^30,31^. Thus, we measured the mRNA and protein expression levels of H-ferritin, L-ferritin, and TfR in the hippocampi of WT and *Prx5*^*−/−*^ mice by using RT-qPCR and western blot analysis. HFe increases H-ferritin and L-ferritin mRNA levels and decreases TfR mRNA levels compared to ND in both WT and *Prx5*^*−/−*^ mice. In addition, H-ferritin and L-ferritin levels are significantly higher in *Prx5*^*−/−*^ mice compared to WT mice with HFe. TfR mRNA levels are lower in *Prx5*^*−/−*^ mice compared to WT mice with HFe (Fig. 1c). The protein levels of TfR, L-ferritin, and H-ferritin showed a similar tendency with mRNA levels (Fig. 1d). We then investigated the iron concentration in hippocampal tissues using an iron assay kit. In WT mice, the iron concentration in hippocampal tissues is slightly increased (not significant; *p* = 0.0613) by HFe compared to ND, while iron concentration is significantly increased by HFe in hippocampal tissues from *Prx5*^*−/−*^ mice (Fig. 1d). Furthermore, to observe hippocampal iron deposits, dissected hippocampi of WT and *Prx5*^*−/−*^ mice were stained with iron stain kit based on the Prussian blue reaction. In *Prx5*^*−/−*^ mice, hippocampal iron content is increased with HFe compared to ND (Fig. 2). These results indicated that Prx5 deficiency aggravates hippocampal iron accumulation under conditions of iron overload.

### Prx5 deficiency exacerbated iron overload-induced ROS production

**Fig. 3:**
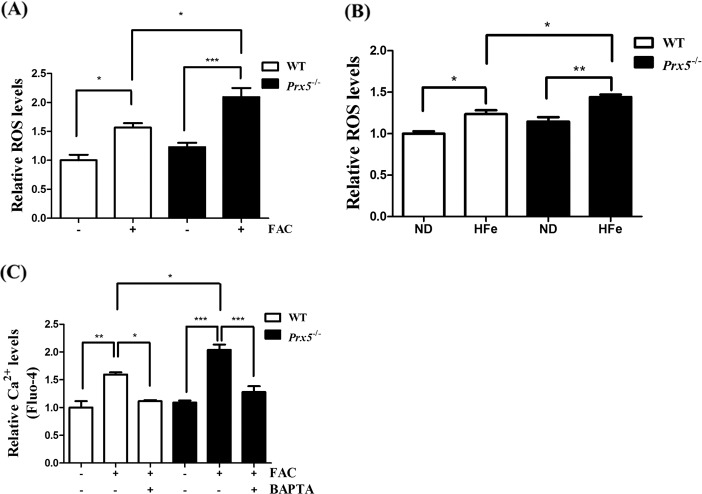
Prx5 deficiency exacerbates iron overload-induced ROS production. **a** Primary hippocampal neurons of WT and *Prx5*^*−/−*^ mice (*n* = 4 per group) were incubated with 150 μM (49.5 μg/mL) FAC for 48 h, and intracellular ROS levels were measured by flow cytometry using CM-H2DCFDA. **b** Relative ROS levels were measured by OxiSelect In Vitro ROS/RNS Assay Kit in hippocampal tissues of WT and *Prx5*^*−/−*^ mice (*n* = 3 per group) with or without HFe. **c** Primary hippocampal neurons of WT and *Prx5*^*−/−*^ mice (*n* = 4 per group) were incubated with FAC for 48 h in the presence or absence of 0.25 μM BAPTA. Relative Ca^2+^ levels were measured by flow cytometry in primary hippocampal neurons of WT and *Prx5*^*−/−*^ mice with Fluo-4 staining. Data are expressed as mean ± SEM of three independent experiments. *p < 0.05, **p < 0.01, and ***p < 0.001.

Because iron overload can generate hydroxyl radicals via Fenton’s reaction, and Prx5 is an antioxidant enzyme, we investigated the effect of Prx5 deficiency on iron overload-induced ROS production in the hippocampus using primary hippocampal neurons and hippocampal tissues. In primary hippocampal neurons of *Prx5*^*−/−*^ mice, ROS levels are significantly increased by 150 μM FAC compared to WT hippocampal neurons (Fig. 3a). Furthermore, ROS levels are increased by HFe in both WT and *Prx5*^*−/−*^ mice. However, the effect is more pronounced in *Prx5*^*−/−*^ mice than in WT mice (Fig. 3b). We also measured the levels of intracellular Ca^2+^ in primary hippocampal neurons by using Fluo-4. Because Fluo-4 can bind Cu^2+^, Fe^2+^ and Zn^2+^ as well as Ca^2+^ ^32^, we used calcium chelator BAPTA to increase confidence regarding to calcium alteration. The results showed that Ca^2+^ levels are increased by FAC in WT and *Prx5*^*−/−*^ primary hippocampal neurons, while BAPTA rescued it (Fig. 3c). Also, the intracellular Ca^2+^ levels are further increased by iron overload in Prx5-deficient hippocampal neurons than in WT hippocampal neurons. These results indicate that *Prx5*^*−/−*^ mice are more susceptible to iron overload than WT mice.

### Prx5 deficiency exacerbates iron overload-induced ER-stress and mitochondrial fission

**Fig. 4:**
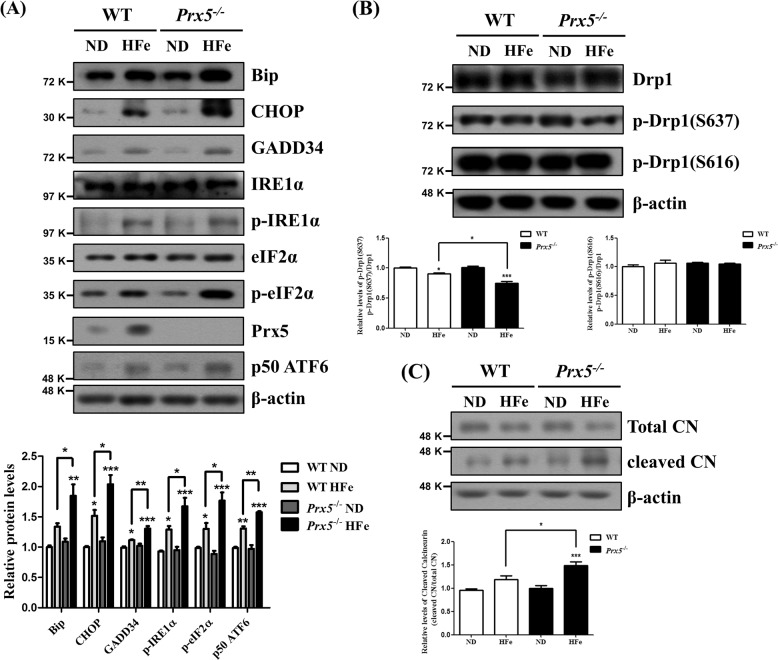
Prx5 deficiency exacerbates iron overload-induced ER-stress and mitochondrial fission. **a** The expression levels of Bip, CHOP, GADD34, IRE1α, phosphorylated IRE1α, eIF2α, phosphorylated eIF2α, ATF6, and Prx5 were determined by western blotting. The graph shows the quantification of protein/β-actin or phosphorylated protein/total protein. **b** The levels of Drp1, phosphorylated Drp1 (Ser637), and phosphorylated Drp1 (Ser616) were assessed by western blotting. The graph shows the quantification of phosphorylated Drp1/Drp1. **c** The level of calcineurin was determined by western blotting. The graph shows the quantification of cleaved calcineurin/total calcineurin. Data are expressed as mean ± SEM of three independent experiments. *p < 0.05, **p < 0.01, and ***p < 0.001.

To examine the effect of Prx5 deficiency on iron overload-induced ER-stress and mitochondrial fission, we investigated markers of ER-stress and mitochondrial dynamics by western blot in hippocampal tissues of WT and *Prx5*^*−/−*^ mice following HFe. The results show that the levels of Bip, CHOP, GADD34, phosphorylated IRE1α, phosphorylated eIF2α, and p50 ATF6 are significantly increased in *Prx5*^*−/−*^ mice compared to WT mice following HFe (Fig. 4a). We also examined expression levels of Drp1, phosphorylated Drp1 (Ser637), and phosphorylated Drp1 (Ser616). As shown in Fig. 4b, only phosphorylated Drp1 (Ser637) levels are decreased by HFe, and this effect is more pronounced in *Prx5*^*−/−*^ mice than in WT mice (Fig. 4b). Because our previous study showed that iron overload-induced dephosphorylation of Drp1 (Ser637) was regulated by calcineurin, which is activated by cleavage^18^, we investigated the expression levels of calcineurin. Our results show that the levels of cleaved calcineurin are significantly increased in *Prx5*^*−/−*^ mice compared to WT mice following HFe (Fig. 4c). These results are consistent with our previous results of iron overload-induced mitochondrial fragmentation in HT-22 cells^18^. Considered together, these findings indicate that Prx5 deficiency exacerbates iron overload-induced ER-stress and Drp1-mediated mitochondrial fission in the hippocampus.

### Prx5 deficiency aggravates iron overload-induced ER-mediated mitochondrial fission in hippocampal primary neurons

**Fig. 5:**
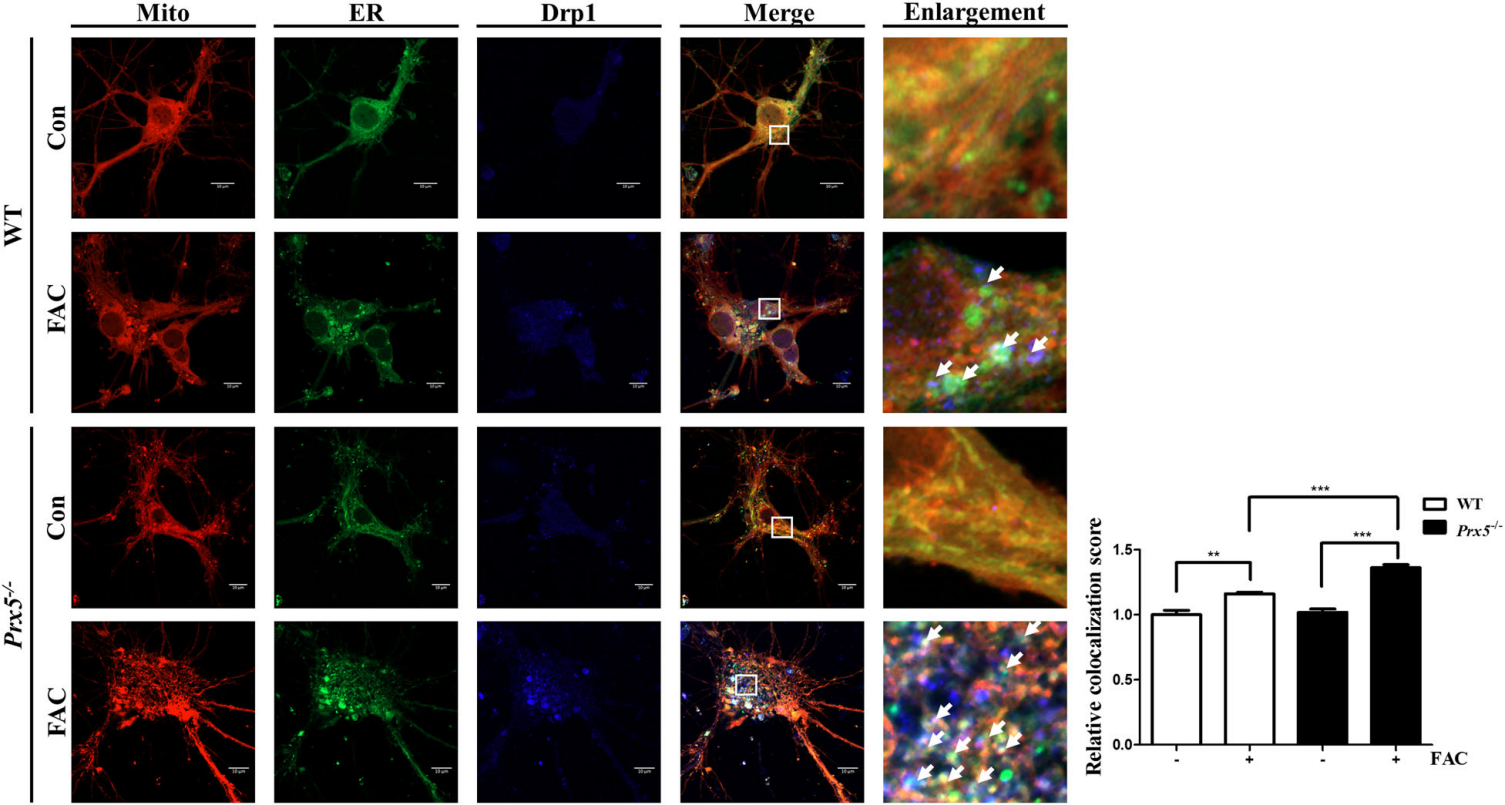
Prx5 deficiency aggravates iron overload-induced ER-mediated mitochondrial fission in primary hippocampal neurons. Primary hippocampal neurons of WT and *Prx5*^*−/−*^ mice (*n* = 3 per group) were cultured with or without 150 μM FAC for 48 h, and then stained with ER-ID (green) and Mito-tracker (red) fluorescent dye after performing immunocytochemistry with anti-Drp1 antibody (blue). The images were obtained by confocal microscopy. White arrows indicate co-localization of the ER, mitochondria, and Drp1 puncta. The fifth panels in each row show magnified images of the regions indicated by white squares in the fourth panels in each row; scale bar = 10 μm. The graph shows the relative colocalization score. Data are expressed as mean ± SEM of three independent experiments. **P < 0.01, ***P < 0.001.

We then investigated the correlation between iron overload-induced mitochondrial fission, ER expansion, and Drp1 localization. To observe Drp1 localization in mitochondria and the ER simultaneously, primary hippocampal neurons of WT and *Prx5*^*−/−*^ mice were stained with Mito-tracker (red) and ER-ID (green) after performing immunocytochemistry using anti-Drp1 antibody. Drp1 localization in mitochondria and the ER were observed using confocal microscopy. Our results show that iron overload increases the co-localization of Drp1 puncta and expanded ER on mitochondria or between fragmented mitochondria in WT hippocampal neurons. Furthermore, Prx5 deficiency shows more co-localization of expanded ER, Drp1 puncta, and mitochondria in the presence of FAC compared to WT hippocampal neurons (Fig. 5). The quantitation of co-localization shows that FAC more increases the co-localization score in *Prx5*^*−/−*^hippocampal neurons than WT hippocampal neurons. These results suggest that iron overload-induced expansion of ER mediates mitochondrial fragmentation via Drp1 translocation in hippocampal neurons, and Prx5 deficiency aggravates this effect.

### Prx5 deficiency exacerbates iron overload-induced hippocampal neuronal death

**Fig. 6:**
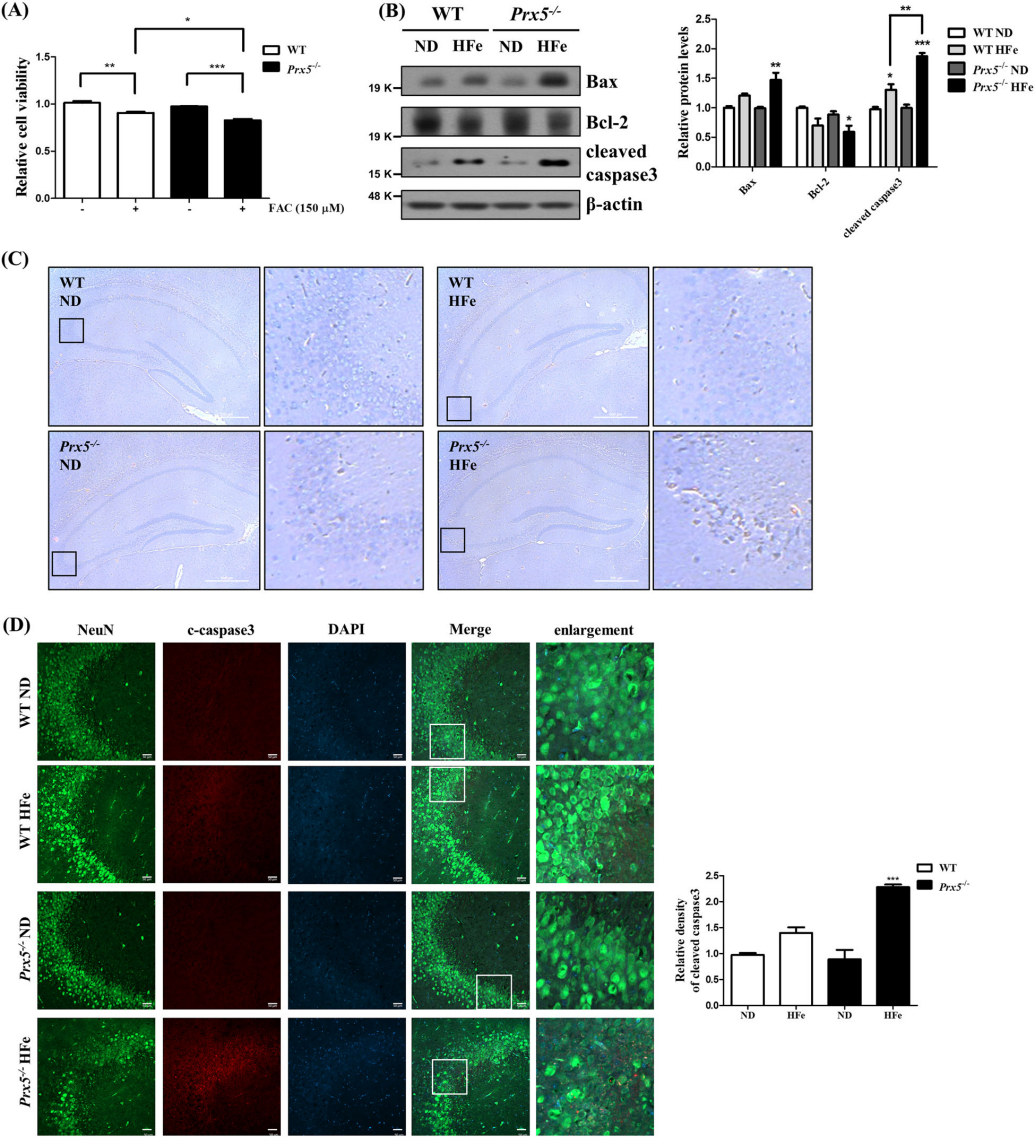
Prx5 deficiency exacerbates iron overload-induced hippocampal neuronal death. **a** Relative cell viability was measured by the MTT assay in FAC-treated primary hippocampal neurons of WT and *Prx5*^*−/−*^ mice (*n* = 4 per group). **b** The levels of Bax, Bcl-2, and cleaved caspase3 were confirmed by western blotting in hippocampal tissues of WT and *Prx5*^*−/−*^ mice (*n* = 3) with or without HFe. The graph shows the quantification of protein/β-actin. **c** Hippocampi of WT and *Prx5*^*−/−*^ (*n* = 3 per group) were observed by H&E staining using an optical microscope. The second and fourth panels in each row show magnified images of the regions indicated by black squares in the first and third panels of each row, respectively; scale bar = 500 μm. **d** Immunohistochemistry images for NeuN (green), cleaved caspase3 (red), and DAPI (blue) were observed by confocal microscopy in hippocampi of WT and *Prx5*^*−/−*^ (*n* = 3 per group) with or without HFe; scale bar = 50 μm. The graph shows the relative density of cleaved caspase3. Data are expressed as mean ± SEM of three independent experiments. **P < 0.01, ***P < 0.001.

Our previous study reported that iron overload induces neuronal death, accompanied by cleavage of caspase3, and Prx5 exerts a protective effect against iron neurotoxicity^18^. Here, we investigate the effects of Prx5 deficiency on iron overload-induced neuronal death in mouse hippocampus. Relative cell viability was measured using the MTT assay in primary hippocampal neurons of WT and *Prx5*^*−/−*^ mice cultured with or without FAC for 48 h. The results show that Prx5 deficiency exacerbates neuronal death in the presence of FAC compared to FAC-treated WT hippocampal neurons (Fig. 6a). We then determined the effect of Prx5 on iron overload-induced changes in apoptotic markers, such as cleaved caspase3, Bax, and Bcl-2 by western blot analysis. The results show that, under iron overload conditions, Prx5 deficiency aggravates iron overload-induced apoptotic cell death in the hippocampus compared to that seen in hippocampal tissues of WT (Fig. 6b). We also observed hippocampal tissues of WT and *Prx5*^*−/−*^ mice by H&E staining. Our results show damage to the CA3 region of the hippocampus in HFe-fed *Prx5*^*−/−*^ mice, while negligible effects are seen in the hippocampus of HFe-fed WT mice (Fig. 6c). We also performed immunohistochemistry with NeuN as a neuronal marker and cleaved caspase3 antibodies in hippocampal tissues of WT and *Prx5*^*−/−*^ mice. The cleaved caspase3-positive neurons are increased by HFe in the CA3 region of both WT and Prx5-deficient hippocampi. However, cleaved caspase3-positive neurons were more frequently detected in Prx5^−/−^ mice compared to WT mice following HFe (Fig. 6d). Furthermore, the localization of cleaved caspase3 to a glial cells was hardly detected in the CA3 region of both WT and Prx5-deficient hippocampi (Supplementary Fig. 2). These results indicate that Prx5 plays an important protective role against iron overload-induced neuronal death in the hippocampus.

### ROS mediates iron overload-induced hippocampal damage

**Fig. 7:**
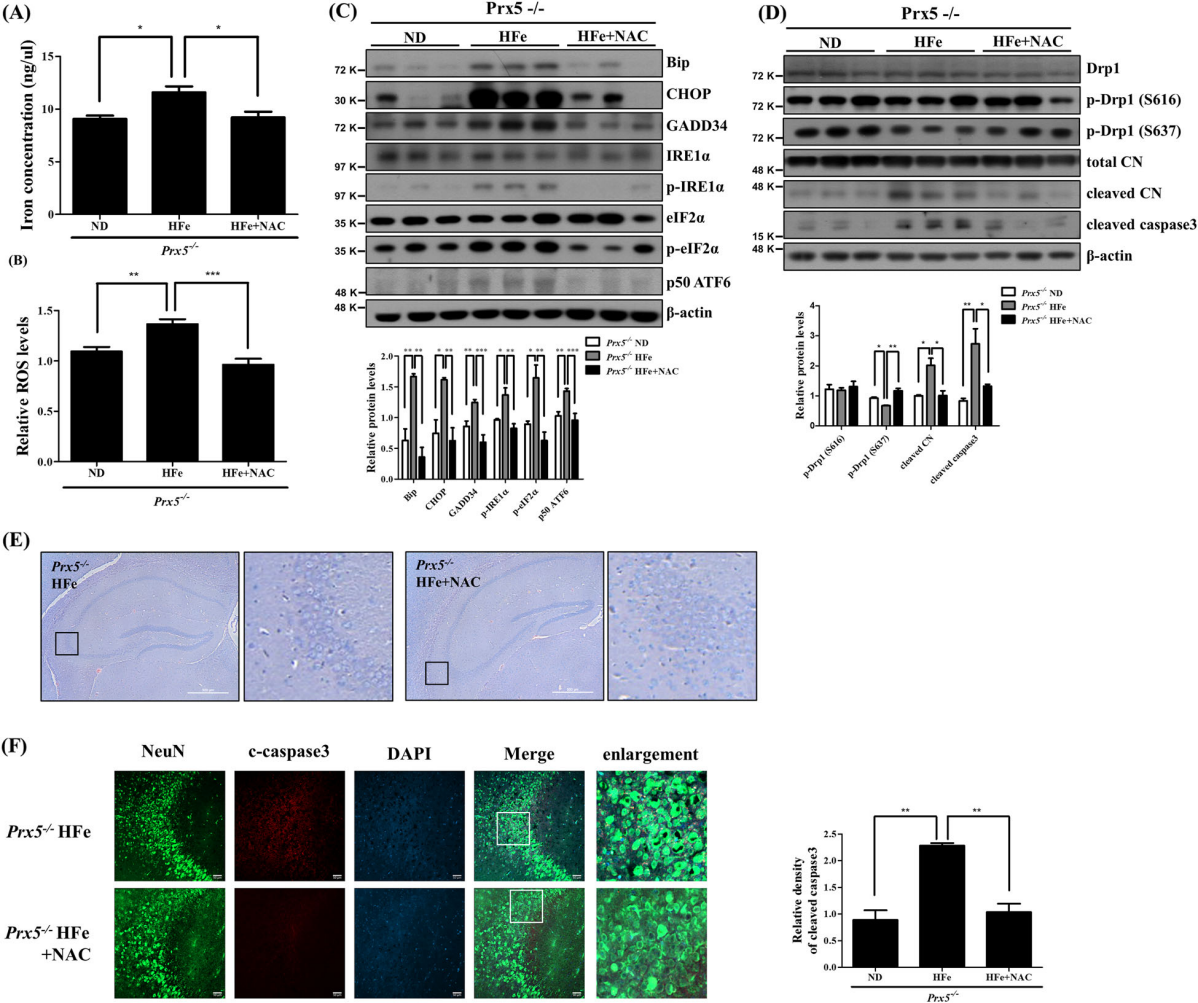
NAC attenuates iron overload-induced hippocampal damage. Iron-loaded *Prx5*^*−/−*^ mice were treated with or without NAC (*n* = 3 per group). **a** The levels of iron concentration were quantified by an iron assay kit in hippocampal tissue. **b** Relative ROS levels were measured by the OxiSelect In Vitro ROS/RNS Assay Kit in hippocampal tissues. **c** The expression levels of Bip, CHOP, GADD34, IRE1α, phosphorylated IRE1α, eIF2α, phosphorylated eIF2α, and ATF6 were determined by western blotting. The graph shows the quantification of proteins/β-actin or phosphorylated protein/total protein. **d** The levels of Drp1, phosphorylated Drp1 (Ser637), phosphorylated Drp1 (Ser616), calcineurin, and cleaved caspase3 were assessed by western blotting. The graph shows the quantification of phosphorylated Drp1/Drp1, cleaved calcineurin/total calcineurin, and cleaved caspase3/β-actin. **e** Hippocampi were observed by H&E staining using an optical microscope. The second and fourth panels in each row show the magnified images of regions indicated by black squares in the first and third panels of each row, respectively; scale bar = 500 μm. **f** Immunohistochemistry images for NeuN (green), cleaved caspase3 (red), and DAPI (blue) were observed by confocal microscopy in hippocampi; scale bar = 50 μm. The graph shows the relative density of cleaved caspase3. Data are presented as mean ± SD (*n* = 3). *p < 0.05, **p < 0.01, and ***p < 0.001.

Because Prx5 deficiency exacerbates iron overload-induced neurotoxicity, we investigated the effect of ROS in iron overload-induced hippocampal damage by measuring neurotoxicity. We investigated whether a reduction in ROS levels can ameliorate iron overload-induced neurotoxicity by using NAC as ROS scavenger. We first assessed the levels of iron concentration and ROS in hippocampal tissues from HFe-fed *Prx5*^*−/−*^ mice with or without NAC. The results show that the increased levels of iron and ROS in the hippocampi of HFe-fed *Prx5*^*−/−*^ mice were alleviated by NAC (Fig. 7a, b). We then confirmed the expression of ER-stress markers, phosphorylated Drp1, calcineurin, and cleaved caspase3 by western blot in HFe-fed *Prx5*^*−/−*^ mice with or without NAC. Our results show that the expression levels of ER-stress markers (Fig. 7c), Drp1 (S637) dephosphorylation, cleaved calcineurin, and cleaved caspase3 (Fig. 7d) were decreased in the NAC-treated group compared to HFe-fed *Prx5*^*−/−*^ mice that did not receive NAC. To further investigate the effect of NAC against iron overload-induced hippocampal damage, we performed H&E staining and immunohistochemistry. In the CA3 region of the hippocampus, NAC treatment attenuated iron overload-induced neuronal loss, and the number of cleaved caspase3-positive neurons returned to normal levels (Fig. 7e, f). These results suggest that Prx5 deficiency aggravates iron overload-induced neurotoxicity by increasing ROS levels.l

## Discussion

Understanding the molecular mechanisms of iron overload-induced neurotoxicity is important because of the critical role that dysregulation of iron homeostasis plays in mediating toxicity and neurodegeneration in several neuronal diseases^33,34^. Iron is important for cellular metabolism processes, including neuronal functions, but excess iron can cause mitochondrial dysfunction and loss of neuronal cells^6,35^. Iron dysregulation, such as iron overload, and oxidative stress have both been associated with mitochondrial dynamics and ER-stress^36–39^. Because iron overload has been involved in oxidative stress via Fenton’s reaction^11^, we hypothesized that iron overload-induced oxidative stress could mediate neurotoxicity related to mitochondrial dynamics and ER-stress. Consequently, we investigated the relationship between iron overload-induced neurotoxicity and ER-stress-mediated mitochondrial fission in mouse hippocampal HT-22 cells^18^. However, in vitro results alone are insufficient to prove the mechanism of iron overload-induced neuronal cell death. In this study, we investigated the mechanism of iron overload-induced neuronal cell death in an in vivo mouse iron overload model using a high iron diet.

The current study suggests that Prx5 plays an important protective role against iron overload-induced hippocampal damage related to ER-stress and mitochondrial fission. Although the glutathione system is well known in the antioxidant defenses of the brain, there is increasing evidence that Prxs also have important roles, including a protective effect in neurological disorders^40–42^. Our previous study showed that iron overload significantly increases the levels of Prx5, while changes in Prx1, Prx2, Prx3, and Prx4 levels are negligible^18^. Our in vivo data of Prx expression levels corresponds with in vitro data that iron overload increases only the levels of Prx5 (Supplementary Fig. 1). Furthermore, Prx5, which is only expressed in the neuron, was uniformly distributed in the CA3 and CA2 regions of the hippocampus, in contrast to the low immunoreactivity of Prx3^43^. Based on these results, we hypothesized that Prx5 has an important role in the hippocampus and investigated the effect of Prx5 deficiency against iron overload-induced hippocampal damage related to oxidative stress and mitochondrial fission in Prx5-knockout mice.

We first confirmed hippocampal iron concentration in WT and *Prx5*^*−/−*^ mice with or without HFe. As shown in Figs. 1 and 2, Prx5 deficiency significantly increased hippocampal iron concentration induced by HFe, while in WT mice, HFe caused a slight increase. Under iron overload conditions, hippocampal ROS levels were increased to higher levels in Prx5-deficient mice than in WT (Fig. 3). These results suggested that Prx5 deficiency makes the hippocampus more sensitive to iron overload. Several studies have indicated that iron regulatory proteins, which are primarily regulated by iron, are also affected by ROS both in vitro and in vivo^44,45^. Therefore, we speculated that excessive ROS levels more induce more iron uptake in *Prx5*^*−/−*^ mice than WT. However, the precise mechanism of the relationship between Prx5 deficiency and increased iron concentration requires further study.

As previously reported, Prx5 exerts a protective effect against ER-mediated mitochondrial fission through calcium/calcineurin/Drp1 pathways in iron-overloaded hippocampal HT-22 cells^18^. The present study also found that iron overload induces ER-stress and ER-mediated mitochondrial fission in the hippocampal tissue of HFe-fed mice and FAC-stimulated hippocampal primary cells, and Prx5 deficiency exacerbates these effects (Figs. 4, 5). Because several studies have shown that iron overload induces apoptosis via activation of caspase3^46,47^, we studied the effect of Prx5 deficiency on iron overload-induced neuronal cell death related to caspase3. As shown in Fig. 6 and Supplementary Fig. 2, Prx5 deficiency aggravated iron overload-induced neuronal cell death in the CA3 region of the hippocampus via caspase3 activation compared to that seen in WT mice, but not in glial cells of CA3 region. It seems that hippocampal neuronal cells are more susceptible to iron overload than glial cells. These results indicated that Prx5 deficiency exacerbates iron overload-induced neuronal cell death via ER-stress and ER-mediated mitochondrial fission by increasing ROS production in hippocampus. To confirm this, we investigated whether an ROS scavenger would rescue hippocampal tissue from iron overload-induced cell death in *Prx5*^*−/−*^ mice. As shown in Fig. 7, treatment with NAC, an ROS scavenger, attenuated iron overload-induced ER-stress and mitochondrial fission and consequently alleviated hippocampal damage. Accordingly, under iron overload conditions, Prx5 deficiency increases ROS-mediated hippocampal cell death through ER-stress and ER/Drp1-dependent mitochondrial fission.

Ferroptosis, which is known as iron-dependent form of programmed cell death, has been well characterized by lipid peroxides accumulation due to the defect of glutathione-dependent antioxidant defenses^48^. Ferroptosis is considered distinct from other forms of cell death such as necrosis and apoptosis. Nevertheless, several studies have reported a cross-talk between ferroptosis and apoptosis, especially related to ER-stress^49,50^. Thus, to investigate the alteration of ferroptosis by iron overload, we assessed ferroptotic markers, such as the levels of GPX activity and lipid peroxidation, in hippocampal tissues from HFe-fed WT and *Prx5*^*−/−*^ mice with or without NAC. Under iron overload conditions, hippocampal GPX activity was decreased to higher levels in Prx5-deficient mice than in WT, and NAC treatment rescued GPX activity to control levels (Supplementary Fig. 3A). In addition, Prx5 deficiency significantly increased hippocampal lipid peroxidation levels induced by HFe compared to WT with HFe, While NAC treatment attenuated it (Supplementary Fig. 3B). These results indicated that Prx5 deficiency also exacerbated ferroptosis in hippocampus, and NAC attenuated it. However, further studies are needed to investigate the precise mechanisms between Prx5 and ferroptosis.

In conclusion, our study is the first reported demonstration that iron overload induces hippocampal cell death via ER-stress and ER-mediated mitochondrial fission in vivo. We found that Prx5 deficiency exacerbates iron overload-induced hippocampal cell death by increasing ROS production. Moreover, we confirmed that Prx5 is relevant to ferroptosis in iron overload-induced hippocampal damage. Based on our previous^18^ and present studies, we suggest that Prx5 has an important role in iron overload-induced hippocampal cell death related to ROS-mediated mitochondrial fission and ferroptosis. We propose that the results of this study provide a basis for possible strategies to develop novel therapies targeting iron overload-associated neuronal disorders.

## Supplementary information

Supplementary Figure legends

Supplementary Figure 1

Supplementary Figure 2

Supplementary Figure 3

author contribution

checklist
