# Supplementary material for: Peroxiredoxin 5 deficiency exacerbates iron overload-induced neuronal death via ER-mediated mitochondrial fission in mouse hippocampus
Source: Cell Death Dis. 2020 Mar 23;11(3):204. doi: 10.1038/s41419-020-2402-7 (PMC7090063; doi:10.1038/s41419-020-2402-7)
Supplement: Supplementary file 1 — Supplementary Figure legends [file 41419_2020_2402_MOESM1_ESM.docx]

**Supplementary Fig. 1.** **Effect of iron overload on expression of Prxs in hippocampal tissue.**

The expression levels of Prx1, Prx2, Prx3, Prx4, Prx5 and Prx6 were determined by western blotting in hippocampal tissues of WT and *Prx5^-/-^* mice with or without HFe. Data are presented as mean ± standard deviation (n = 3). ***p < 0.001.

**Supplementary Fig. 2.** **Immunohistochemistry in CA3 region of hippocampal tissues.**

Immunohistochemistry images for GFAP (green), cleaved caspase3 (red), and DAPI (blue) were observed by confocal microscopy in hippocampi of iron-loaded WT and *Prx5^-/-^* with or without NAC; scale bar = 50 μm.

**Supplementary Fig. 3.** **Effect of iron overload on ferroptotic markers in hippocampal tissue.**

(A) GPX activity and (B) lipid peroxidation were measured using Glutathione Peroxidase Assay Kit (Cayman, MI, USA) and TBARS Assay Kit (Cayman), respectively, in Hippocampal tissues of iron loaded of WT and *Prx5^-/-^* mice with or without NAC. Data are presented as mean ± standard deviation (n = 3). *p < 0.05, **p < 0.01, and ***p < 0.001.
