# Supplementary figures and images for: Peroxiredoxin 5 deficiency exacerbates iron overload-induced neuronal death via ER-mediated mitochondrial fission in mouse hippocampus
Source: Cell Death Dis. 2020 Mar 23;11(3):204. doi: 10.1038/s41419-020-2402-7 (PMC7090063; doi:10.1038/s41419-020-2402-7)

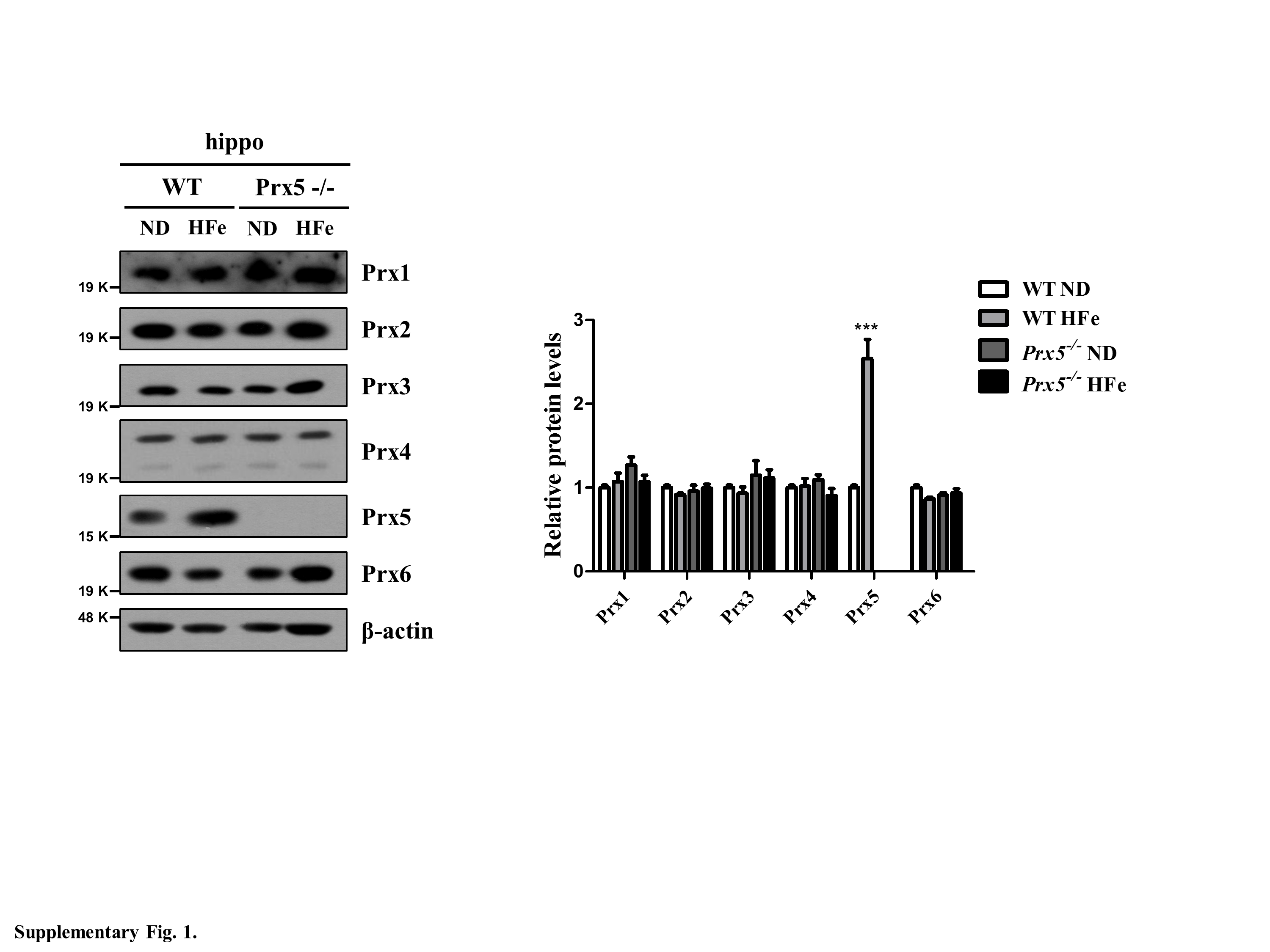

Supplement: Supplementary file 2 — Supplementary Figure 1 [file 41419_2020_2402_MOESM2_ESM.tif]

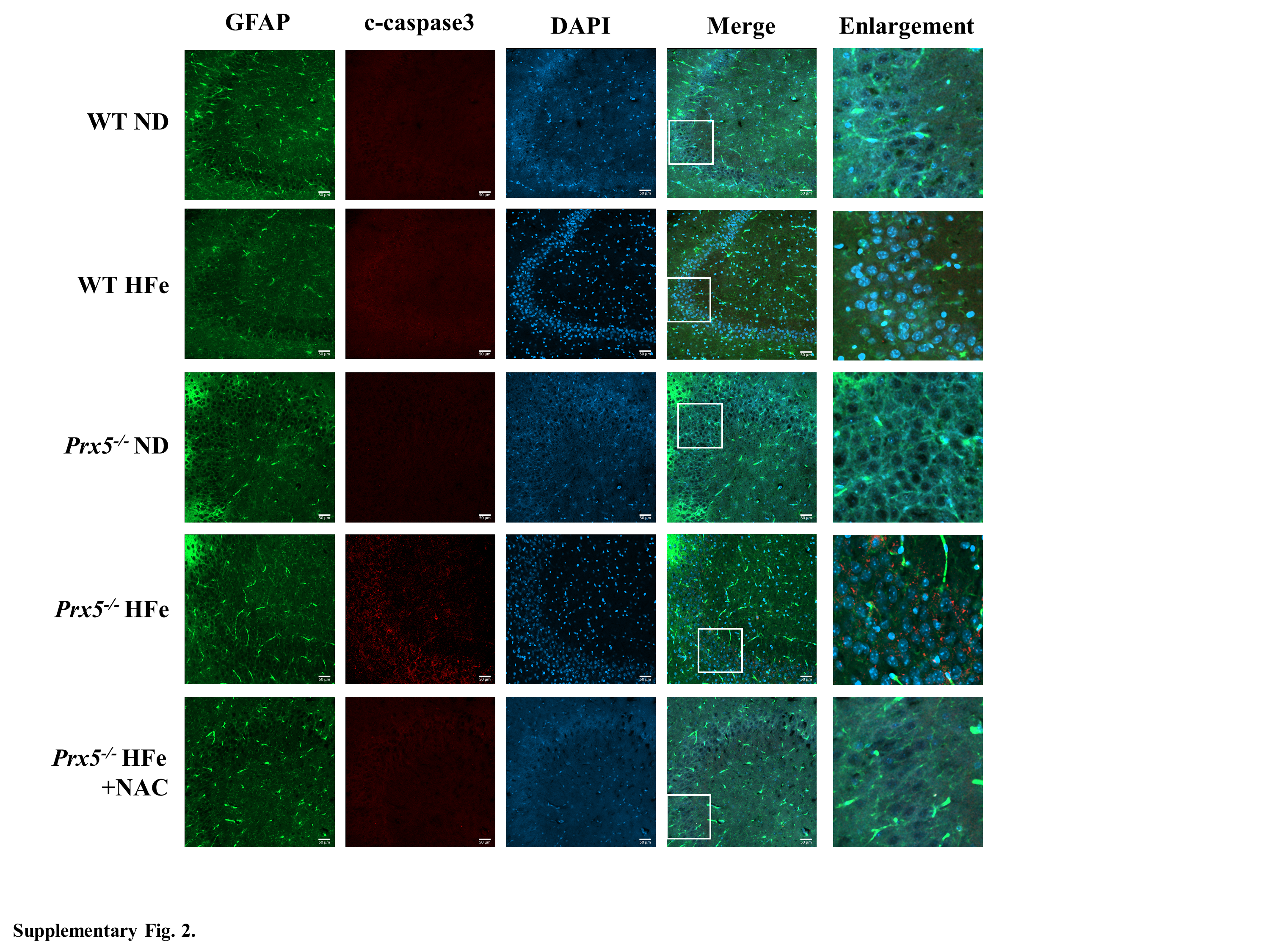

Supplement: Supplementary file 3 — Supplementary Figure 2 [file 41419_2020_2402_MOESM3_ESM.tif]

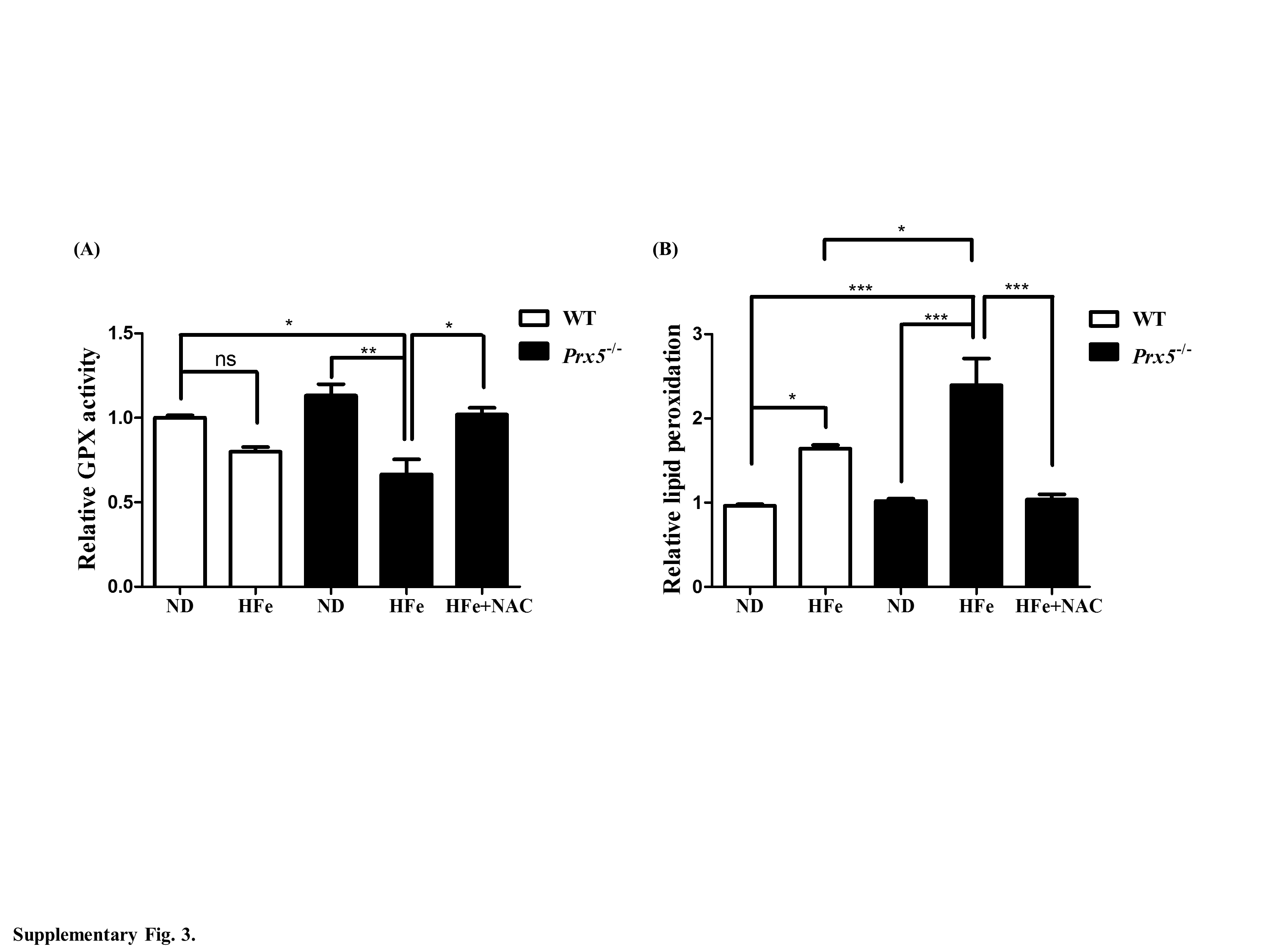

Supplement: Supplementary file 4 — Supplementary Figure 3 [file 41419_2020_2402_MOESM4_ESM.tif]
