# Supplementary material for: Peroxiredoxin 5 deficiency exacerbates iron overload-induced neuronal death via ER-mediated mitochondrial fission in mouse hippocampus
Source: Cell Death Dis. 2020 Mar 23;11(3):204. doi: 10.1038/s41419-020-2402-7 (PMC7090063; doi:10.1038/s41419-020-2402-7)
Supplement: Supplementary file 5 — author contribution [file 41419_2020_2402_MOESM5_ESM.pdf]

DECLARATION OF CONTRIBUTIONS TO ARTICLE

ADMC

Manuscript Number: CDDIS-19-3282R

Journal Name: *Cell Death & Disease* (the 'Journal')

Proposed Title of the Contribution:

Peroxiredoxin 5 deficiency exacerbates iron overload-induced neuronal death [illegible] (the 'Contribution)

Author(s):

Dong Gil Lee, Min Kyoung Kam, Sang-Rae Lee, Hong Jun Lee, Dong-Seok Lee (the 'Authors')

For all *CDDis* articles, each person named as an author in the published version must be able to show he or she has contributed substantially to the article.

Authorship credit should be based on 1) substantial contributions to conception and design, acquisition of data, or analysis and interpretation of data; 2) drafting the article or revising it critically for important intellectual content; and 3) final approval of the version to be published. Authors should meet conditions 1, 2 and 3.

Any person who cannot be shown to have made a substantial contribution to the article cannot be listed as an author in the final version. The name of any person who is deemed to have made a minor contribution can, however, appear in the Acknowledgments section of the article.

Please complete the table below to indicate the contributions of all named authors to the manuscript.

Author Full Name:	Specification of Contribution to the Manuscript:
Dong Gil Lee	designed the study, performed the in vivo experiments
Min Kyoung Kam	performed the primary culture, western blot
Sang-Rae Lee	data analysis, financial support
Hong Jun Lee	analyzed and interpreted data
Dong-Seok Lee	designed the study, wrote the manuscript, approved the [illegible]

Please complete the table below to indicate the contributions of all named authors to the figures.

Figure 1:

Dong Gil Lee, Sang-Rae Lee

Figure 2:

Dong Gil Lee, Hong Jun Lee

Figure 3:

Dong Gil Lee, Min Kyoung Kam

Figure 4:

Dong Gil Lee, Min Kyoung Kam

Figure 5:

Min Kyoung Kam, Dong-Seok Lee

Figure 6:

Dong Gil Lee, Dong-Seok Lee

Signed for and on behalf of the Author(s):	Print Name:	Date:
	Dong-Seok Lee	February 1, 2020
